# Comparison of LC–MS/MS and CLIA Methods for Vitamin D Measurement in an Unselected Outpatient Cohort: Statistical Evaluation and Impact on Clinical Classification

**DOI:** 10.3390/diagnostics16162514

**Published:** 2026-08-09

**Authors:** Carlo Corbetta, Luigi Corsaro, Eugenio Caradonna, Chiara Rusconi, Ivana De Rosa, Stefano Diani, Veniero Gambaro, Ugo de Grazia, Alessandro Maiocchi, Fulvio Ferrara, Lucy Costantino, Katia Roda

**Affiliations:** 1 Laboratory Research Unit, CDI—Centro Diagnostico Italiano S.p.A., Via Saint Bon 20, 20147 Milan, Italy; carlo.corbetta@cdi.it (C.C.); eugenio.caradonna@cdi.it (E.C.); 2 Clinical Pathology Laboratory, CDI—Centro Diagnostico Italiano S.p.A., Via Saint Bon 20, 20147 Milan, Italy; chiara.rusconi@cdi.it (C.R.); ivana.derosa@cdi.it (I.D.R.); stefano.diani@cdi.it (S.D.); veniero.gambaro@cdi.it (V.G.); ugo.degrazia@cdi.it (U.d.G.); katia.roda@cdi.it (K.R.); 3 Medical Genetic Laboratory, CDI—Centro Diagnostico Italiano S.p.A., Via Saint Bon 20, 20147 Milan, Italy; lucy.costantino@cdi.it; 4 Department of Pharmaceutical Science, Università Degli Studi di Milano, Via Luigi Mangiagalli 25, 20133 Milan, Italy; 5 Bracco S.p.A., Via Egidio Folli 50, 20134 Milan, Italy; alessandro.maiocchi@bracco.com; 6 Integrated Laboratory Medicine Service, CDI—Centro Diagnostico Italiano S.p.A., Via Saint Bon 20, 20147 Milan, Italy; fulvio.ferrara@cdi.it

**Keywords:** 25-hydroxyvitamin D, assay comparison, chemiluminescence immunoassay, clinical reclassification, liquid chromatography–mass spectrometry

## Abstract

Background: This study aimed to compare a chemiluminescence immunoassay (CLIA; Siemens Atellica IM 1600) with an automated liquid chromatography–tandem mass spectrometry (LC–MS/MS) analyser (Thermo Scientific Cascadion SM) for serum 25-hydroxyvitamin D [25(OH)D] measurement in unselected outpatients, and its impact on clinical classification. Methods: 25(OH)D was measured on 1064 paired serum samples (828 women, 236 men; age 3–111 years); both platforms participated in the Vitamin D Standardisation Program (VDSP). Agreement was assessed by the Wilcoxon signed-rank test, Passing–Bablok regression, Bland–Altman analysis on log-transformed values, Breusch–Pagan testing for heteroscedasticity, consistency-based intraclass correlation coefficient (ICC), Lin’s concordance correlation coefficient, and Cohen’s kappa with McNemar’s test on six- and two-category clinical classifications. Results: Methods differed significantly ($p<2.2\times 10^{-16}$). Passing–Bablok regression showed proportional bias (slope 1.33; 95% confidence interval [CI], 1.28–1.38; LC–MS/MS higher). Consistency ICC was 0.928 (95% CI, 0.919–0.936). Log-Bland–Altman bias was 35.5% (limits of agreement, −18.3% to +124.5%; heteroscedasticity, $p=5.18\times 10^{-11}$). Cohen’s kappa was 0.32 unweighted and 0.72 weighted on six clinical categories, and 0.56 on a two-class scheme (McNemar $p<2.2\times 10^{-16}$). In discordant pairs, CLIA classified deficiency 118-fold more often than LC–MS/MS. Conclusions: In our study, despite preserved rank ordering, a non-linear concentration-dependent bias and different limits of agreement caused systematic differences in the classification of vitamin D deficiency. Adoption of automated LC–MS/MS can substantially reclassify outpatients toward sufficiency, with implications for supplementation and laboratory harmonisation.

## 1. Introduction

The term “vitamin D” is a misnomer, as this molecule is a hormone [1], specifically a secosteroid, synthesised endogenously via UVB-mediated conversion of 7-dehydrocholesterol in the skin [2]. Vitamin D status results from this endogenous synthesis combined with dietary intake of vitamin D_3_ (animal) and D_2_ (plant). The hormone regulates calcium/phosphorus metabolism, binds to Vitamin D-Binding Protein (VDBP), and interacts with Vitamin D Receptor (VDR) in multiple tissues, mediating diverse systemic effects [3,4].

Recent advances have elucidated metabolites, enzymatic reactions, transport proteins, receptors, and genomic actions, highlighting classical (skeletal) and pleiotropic (extraskeletal) effects, including immunomodulation, metabolic regulation, and links to obesity, diabetes, cardiovascular, neurocognitive disorders, cancer, and viral infections such as SARS-CoV-2 [2,5,6,7,8].

Epidemiologically, vitamin D deficiency is widespread globally, affecting all ages and sexes, and is considered a potential public health problem [9,10,11,12,13,14,15]. Although routine screening in asymptomatic adults is not recommended [16], assessment is crucial for at-risk populations. In Italy, the Italian Medicines Agency (AIFA) defines eligibility criteria for vitamin D prescriptions covered by the National Health Service [17,18,19].

Measurement of vitamin D has evolved from radioimmunological and chromatographic, to immunometric and LC–MS/MS methods, though automated immunometric methods remain prevalent [20,21]. The Vitamin D External Quality Assessment Scheme (DEQAS) in the 2016/2017 review remarks that 79% of laboratories used immunometric methods, 19% LC–MS/MS [22].

Vitamin D status is assessed by Total 25(OH)D, comprising $25\left(\mathrm{OH}\right)D_{3}$ and $25\left(\mathrm{OH}\right)D_{2}$, which reflects dietary intake, cutaneous synthesis, and hepatic metabolism [14,23,24]. It is preferred over the active metabolite $1,\;25{\left(\mathrm{OH}\right)}_{2}D$ due to higher stability, longer half-life, and accurate reflection of body stores [15,25].

Accurate quantification remains complex due to simultaneous measurement of D_3_ and D_2_ forms, other circulating vitamin D metabolites, protein binding, and interferences in certain conditions (renal disease, pregnancy, drug therapy) [26,27,28]. Automated immunometric methods meet demand but show high inter-method variability [29,30]. The difficulties in accurately defining individual vitamin D status led, in 2010, to the promotion of the worldwide Vitamin D Standardization Program (VDSP) by some international institutions: the U.S. National Institutes of Health (NIH-ODS), National Institute of Standards and Technology (NIST), Centers for Disease Control and Prevention (CDC), and Ghent University (Belgium) [31]. The VDSP defined reference procedures, materials, and certification programs to standardise assays and ensure precision and also minimum acceptability criteria for analytical variability: coefficient of variation (CV%) < 10% and mean bias <+5% [24,31,32].

A further important contribution to the standardisation of vitamin D measurement methods has been provided by the Vitamin D External Quality Assessment Scheme (DEQAS), a proficiency testing programme launched in 1989 and progressively developed into an accuracy-based scheme through the use of a Reference Measurement Procedure (RMP), initially established by the National Institute of Standards and Technology (NIST) and subsequently by the Centers for Disease Control and Prevention (CDC). As comprehensively summarised by Briggs et al. [33], DEQAS is the foremost international external quality assessment (EQA) programme for the measurement of 25-hydroxyvitamin D and other vitamin D metabolites, with more than 500 participants across 40 countries. The scheme operates through the quarterly distribution of serum samples to participants, each assigned a target value by means of the aforementioned reference procedures, thereby enabling participating laboratories to assess the accuracy of their own methods against a “gold-standard” RMP.

In 2021, the CDI Laboratory Medicine Department implemented an automated LC–MS/MS system (Thermo Scientific Cascadion SM Clinical Analyser) for vitamin 25(OH)D measurement, transitioning from immunometric methods after validation, with routine use from May 2021 to December 2022 [34]. The present study analyses the results obtained in the initial phase of comparison between the immunoassay method in use and the new method in LC–MS/MS.

## 2. Materials and Methods

### 2.1. Analytical Platforms and Samples

Two platforms were compared:

Siemens Atellica IM 1600 (Siemens Healthineers, Erlangen, Germany) is a high-throughput immunoassay analyser (up to 440 tests/h) using acridinium ester chemiluminescence, integrated within the Atellica Solution system. Total 25-hydroxyvitamin D was measured using the competitive Atellica^®^ IM Vitamin D Total (VITD) assay with a two-level calibration and measuring range 4.2–150 ng/mL [35].

Thermo Scientific^®^ Cascadion^®^ SM Clinical Analyser (Thermo Fisher Scientific, Vantaa, Finland) is a fully automated LC–MS/MS platform integrating centrifugation, sample preparation, dual LC systems, and a triple-quadrupole mass spectrometer. The system quantifies $25\left(\mathrm{OH}\right)D_{2}$ and $25\left(\mathrm{OH}\right)D_{3}$, calculating sum as Total 25(OH)D, excluding C3 epimers. The Cascadion^®^ SM 25-Hydroxy Vitamin D Assay contains ready-to-use reagents that include an internal standard, six-level calibrators, and three-level QC materials, with an analytical range of 3.6–115.9 ng/mL for $25\left(\mathrm{OH}\right)D_{2}$ and 4.2–121.4 ng/mL for $25\left(\mathrm{OH}\right)D_{3}$.

Both platforms followed manufacturer calibration procedures. The Cascadion platform used six-level calibrators (single batch) for both measurands, and three-level kit QC materials for internal quality control (IQC). The Atellica platform used two-level calibrators (single batch) and third-party IQC materials (Table 1, Table 2, Table 3, Table 4 and Table 5, reporting mean, SD, and CV%).

Serum samples were analysed within 24 h of collection. Primary tubes were centrifuged within 30 min of sampling and stored at 4–8 °C until analysis. Testing was prioritised on the Atellica IM 1600 platform, while the Cascadion platform was operated in an overnight analytical cycle.

### 2.2. Study Population

Between 22 February and 9 April 2021, the CDI Central Laboratory received 72,742 laboratory requests: 41,639 (57.24%) from females and 31,103 (42.76%) from males, aged 3–111 years. Measurement of Total Vitamin 25(OH)D was requested in 12,404 cases (17.05%): 9704 females (78.23%) and 2700 males (21.77%). Demographics of the total laboratory population are summarised in Table 6 and Table 7.

**Figure 1:**
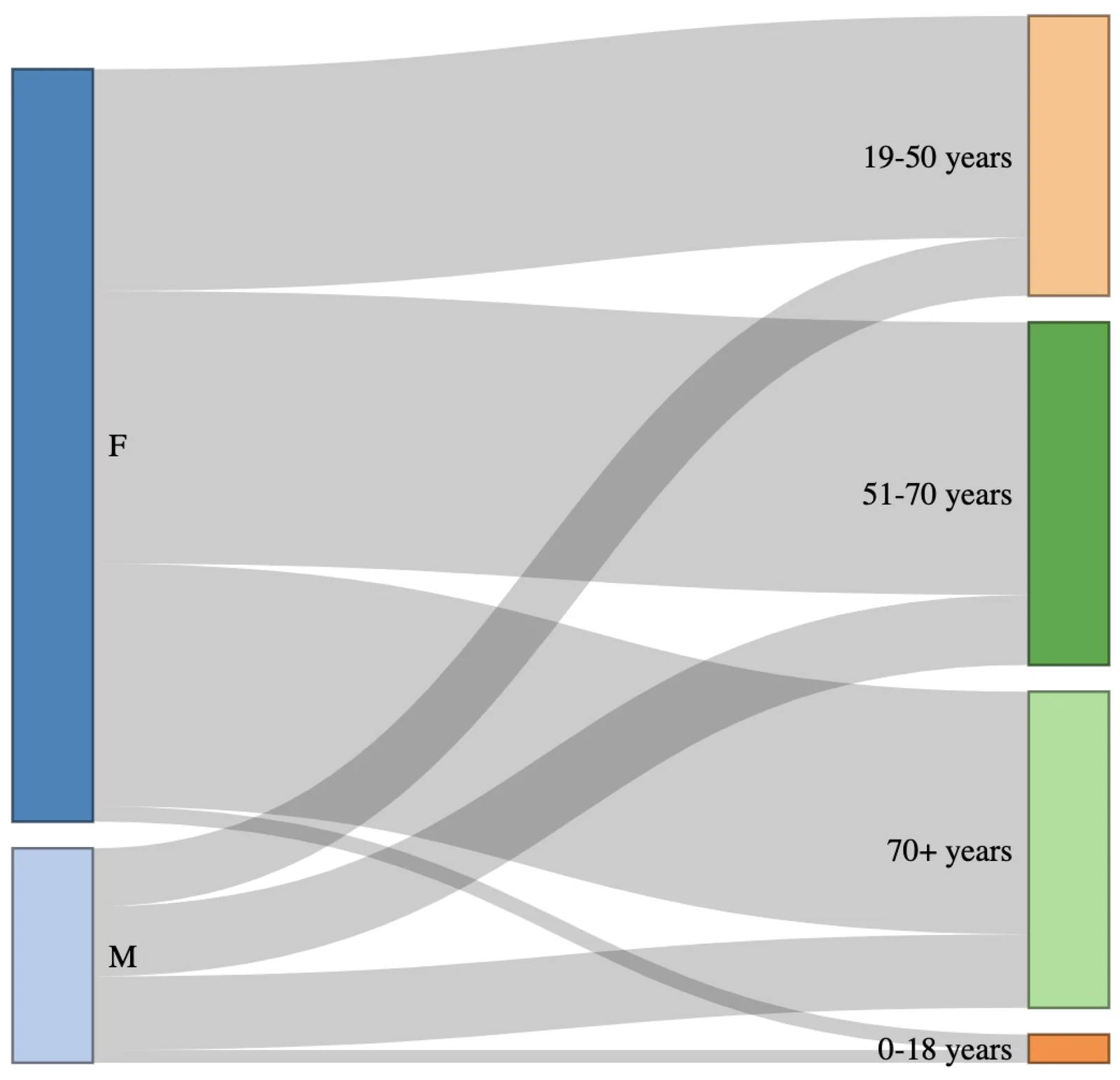
Distribution of selected samples by age group and sex.

A subset of 1064 samples (8.6%) was selected for Total Vitamin 25(OH)D measurement (828 females, 236 males), reflecting the original population (Figure 1).

Characteristics of the selected sub-cohort are summarised in Table 8.

### 2.3. Statistical Analysis

#### 2.3.1. Sample Size

The minimum sample size was estimated for a paired-design comparison of the two methods. A clinically relevant between-method difference of 2 ng/mL was assumed, corresponding to exactly 10% of the 20 ng/mL deficiency decision threshold, consistent with the total allowable error commonly adopted for 25(OH)D and with method-comparison recommendations (CLSI EP09) [36]. The standard deviation of the paired differences (3 ng/mL) was derived from the inter-/intra-laboratory coefficients of variation reported for standardised 25(OH)D assays [32]. With a two-sided significance level α=0.01 and 99% power in a paired *t* framework, the required size was 58 paired samples (rounded to 60). To accommodate pre-specified stratified analyses across eight sex- and age-based subgroups, accounting for male under-representation (ratio ≈ 1:3), the target was set at 960 paired samples. In practice, 1064 paired samples were collected over 17 working days, exceeding the calculated requirement. Stratification by sex and age was pre-specified because inter-method discordance in 25(OH)D measurement has been reported to vary with these demographic factors [37], so verifying the homogeneity of the between-method bias across strata was a planned sensitivity analysis rather than a post hoc exploration.

#### 2.3.2. Analysis Methods

Agreement and consistency between methods were assessed using the following:

- Wilcoxon signed-rank test for paired data: Used to assess whether the two methods provided significantly different median values in the same subject, stratified by age and sex. Normality of the paired between-method differences (Immunoassay − LC–MS/MS) was first assessed by the Shapiro–Wilk test; as the differences departed significantly from normality, the non-parametric Wilcoxon signed-rank test was adopted, a paired *t*-test being inappropriate.
- Passing–Bablok linear regression: Applied to describe the relationship between the values obtained with the two methods, estimating potential systematic bias (constant and proportional).
- Bland-Altman analysis: Performed on log-transformed values to assess systematic bias and evaluate limits of agreement across the measurement range. Heteroscedasticity was formally tested using the Breusch-Pagan test applied to the classical (untransformed) Bland-Altman residuals.
- Intraclass Correlation Coefficient (ICC): Calculated as ICC(C,1) for consistency—to quantify rank-order preservation between the two methods, independently of systematic bias.
- Concordance Correlation Coefficient (CCC): Used to evaluate absolute agreement, combining in a single measure both precision (how close the observations are to each other) and accuracy (how close they are to the line of identity).
- Cohen’s Kappa and McNemar’s test: Applied to assess whether the two methods yielded discordant clinical classifications of vitamin D status (insufficiency vs. sufficiency). A confusion matrix was constructed and the discordance ratio calculated; analyses were restricted to these two categories to preserve statistical power. McNemar’s test evaluated the significance of discordant pairs.

All analyses were conducted at a significance level of α=0.01 in R version 4.6.0 with the packages mcr (version 1.3.3.1), psych (version 2.6.5), and irr (version 0.85).

## 3. Results

### 3.1. Analytical Results

Total 25(OH)D measurements are shown by sex and platform in Table 9 and Table 10. Both methods show higher Total 25(OH)D median in females (CLIA: $p=1.945\times 10^{-6}$, LC–MS/MS: $p=5.8\times 10^{-11}$).

### 3.2. Method Comparison

**Figure 2:**
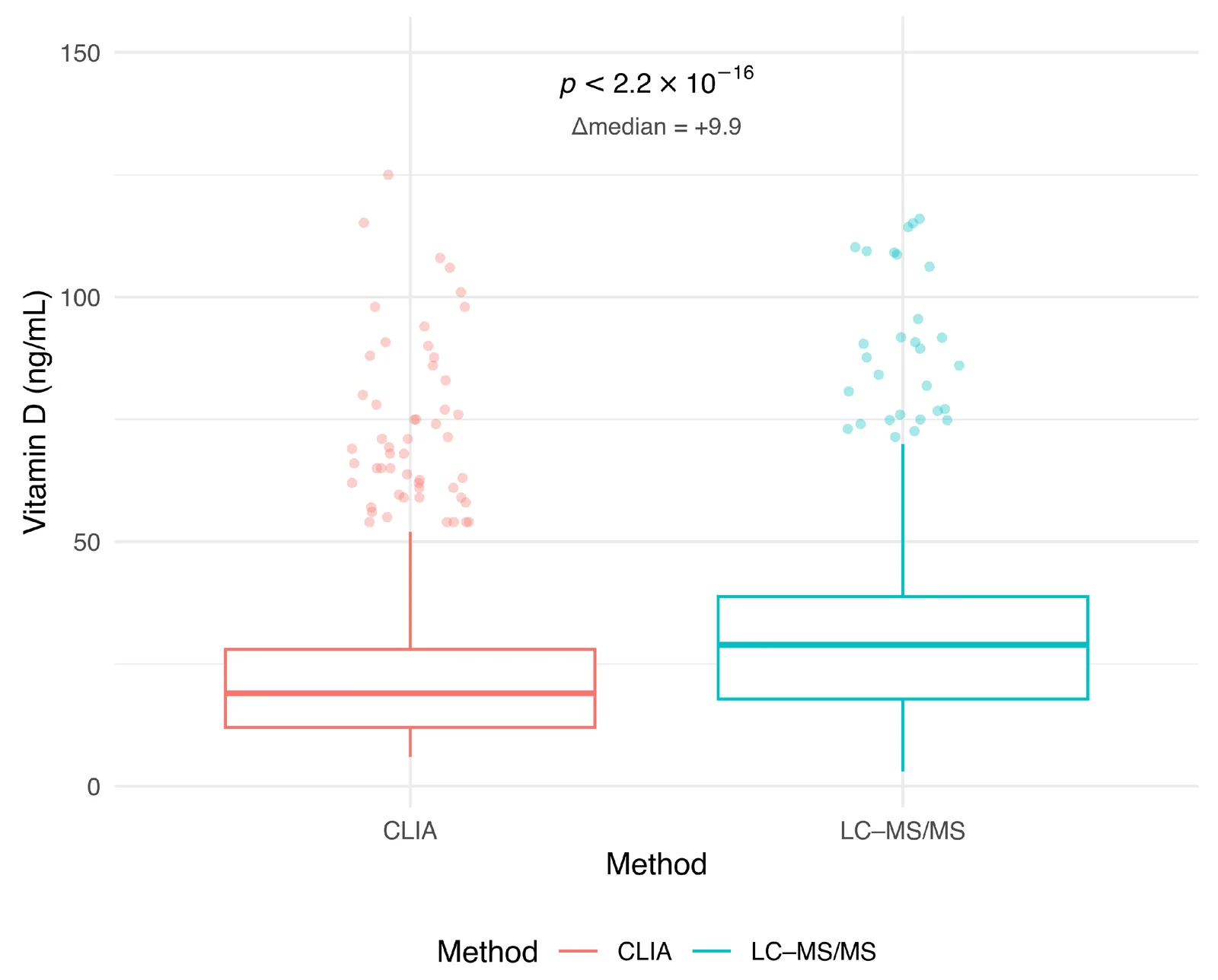
Comparison of methods across the total study population (*n* = 1064). Each box shows the median and interquartile range of the values returned by one method over the whole cohort, with whiskers extending to 1.5 times the interquartile range. The dots above the whiskers are the individual values falling outside that range; they are spread horizontally so that overlapping samples remain distinguishable, and their vertical position is unchanged. The paired Wilcoxon *p*-value and the difference between the two medians are reported above the boxes.

**Figure 3:**
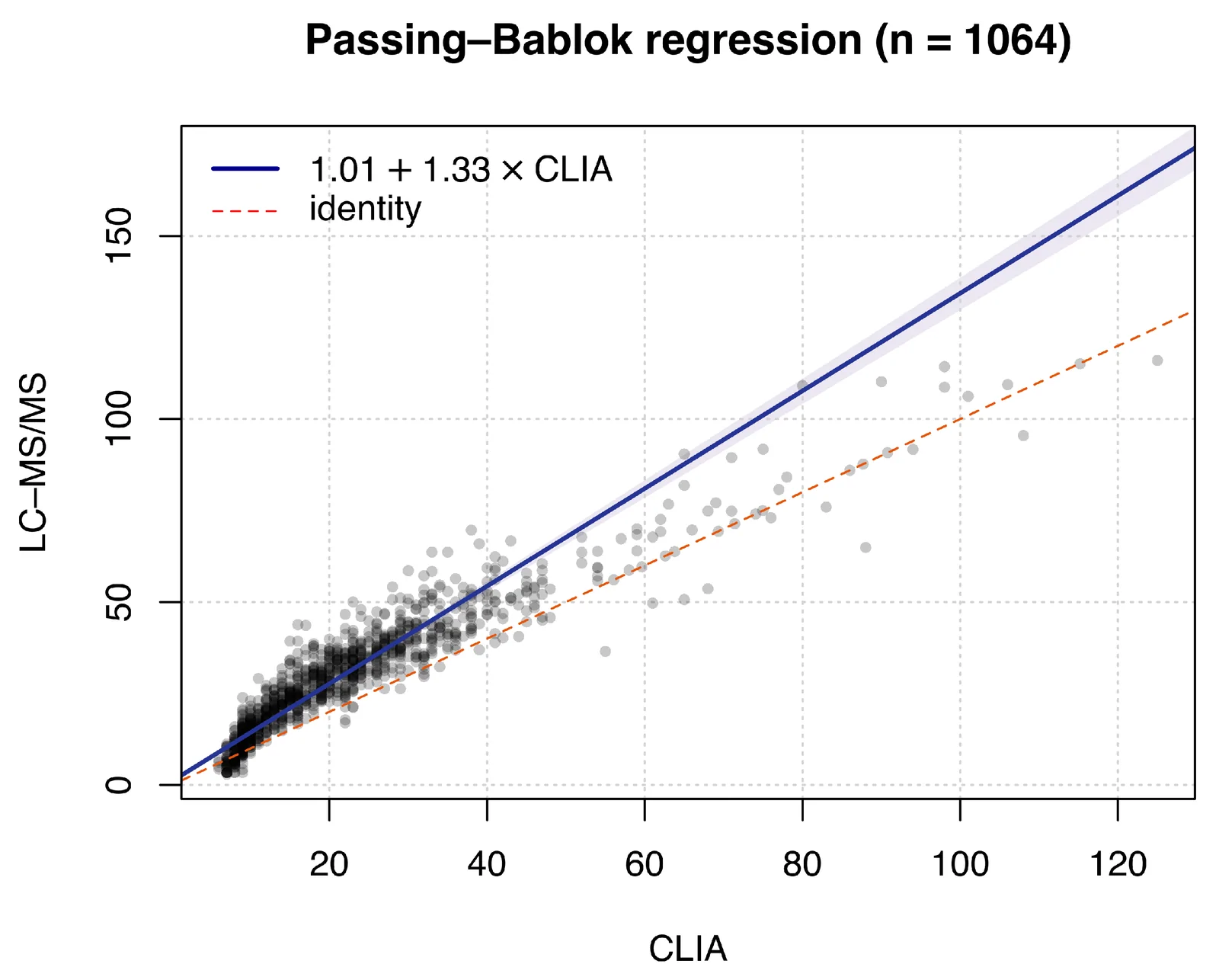
Passing–Bablok regression comparing the two measurement methods over the whole cohort (*n* = 1064). Each grey dot is a single patient sample measured by both methods: its position along the horizontal axis is the value returned by CLIA, and its position along the vertical axis is the value returned by LC–MS/MS. The dots are semi-transparent, so that where many samples fall close together the shade appears darker. The solid blue line is the Passing–Bablok regression fitted to these points, and its equation is reported in the legend inside the plot; the pale band around the line is its 95% confidence interval, obtained by bootstrap. The dashed orange line is the line of identity, that is, the position the points would occupy if the two methods returned the same value.

The Wilcoxon test confirmed quantitative differences ($p=2.2\times 10^{-16}$, Figure 2). Passing–Bablok regression (Figure 3) showed a slope of 1.33 (95% CI: 1.28–1.38) and an intercept of 1.01 (95% CI: 0.09–1.86). With the CLIA method as (*x*) and the LC–MS/MS method as (*y*), the slope significantly greater than 1 indicates a proportional bias: the LC–MS/MS method systematically yields higher values than the CLIA method, with the difference increasing proportionally with measurement magnitude.

Given the proportional bias identified by Passing–Bablok regression, absolute agreement between methods is not expected a priori; accordingly, consistency-based ICC is the appropriate metric to assess whether the two methods preserve the same rank ordering across subjects. ICC(C,1) = 0.928 (95% CI: 0.919–0.936) indicates high consistency, confirming strong rank preservation despite the systematic bias.

**Figure 4:**
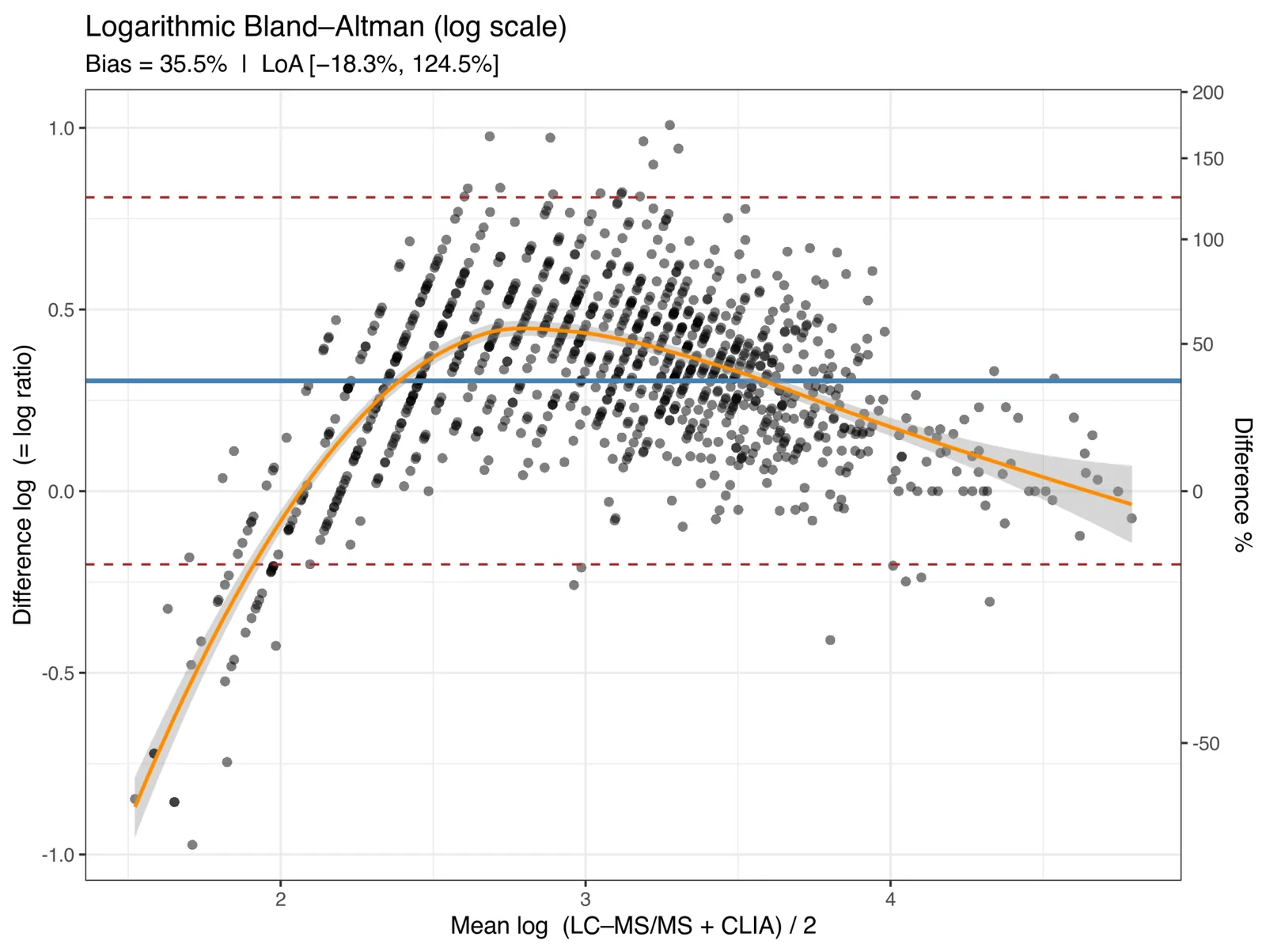
Bland–Altman analysis of the two methods on log-transformed values (*n* = 1064). Each dot is a single patient sample: its position along the horizontal axis is the mean of the two log-transformed measurements, and its position along the vertical axis is the difference between them. All dots are drawn in the same colour and are semi-transparent, so that where many samples overlap the shade appears darker; no colour coding is intended. The left-hand axis reports the difference on the logarithmic scale, that is, the logarithm of the ratio between the two methods, while the right-hand axis reports the same difference expressed as a percentage. The solid blue horizontal line is the mean bias between the methods, and the two dashed red horizontal lines are the upper and lower limits of agreement; both values are also reported above the plot. The orange curve is a locally weighted (LOESS) smoother of the differences, which describes how the difference between the methods varies across the measurement range, and the grey band around it is its 95% confidence interval.

Bland–Altman analysis performed on log-transformed values revealed a mean bias of 35.5% (LoA (limits of agreement): −18.3% to +124.5%; Figure 4), confirming systematic discrepancy across the entire measurement range. These limits of agreement are potentially relevant for vitamin D measures, where clinical decision thresholds differ by as little as 10–20 ng/L. Importantly, log transformation did not resolve heteroscedasticity (Breusch–Pagan $p=5.18\times 10^{-11}$). The LOESS (Locally Extimated Scatterplot Smoothed) trend on the log-scale Bland–Altman plot follows a non-linear trajectory—increasing steeply at low concentrations and decreasing at higher concentrations—indicating that the discrepancy between methods does not follow a simple proportional structure. This concentration-dependent behaviour limits the effectiveness of standard log or regression-based corrections and further undermines method interchangeability. The clinical implications of this non-constant bias are examined in Section 4.

**Figure 5:**
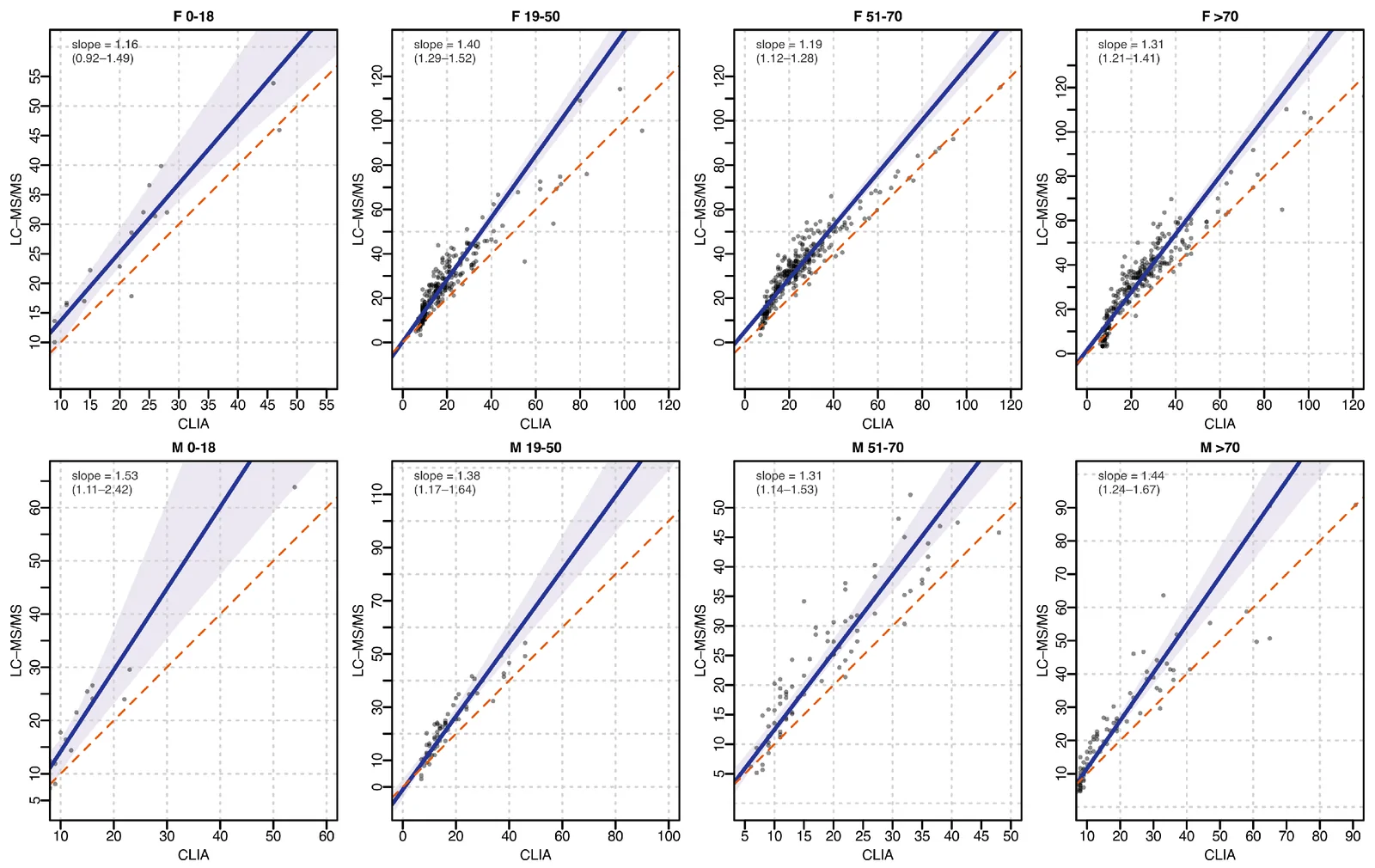
Comparison of the two methods within each sex and age stratum, by Passing–Bablok regression. Each panel corresponds to one stratum, indicated in the panel heading (F, female; M, male; age in years), and is built in the same way as Figure 3: each grey dot is a single patient sample, placed according to its CLIA value on the horizontal axis and its LC–MS/MS value on the vertical axis; the solid blue line is the Passing–Bablok regression fitted within that stratum, the pale band around it is its 95% confidence interval obtained by bootstrap, and the dashed orange line is the line of identity. The value printed at the top left of each panel is the slope of the Passing–Bablok regression for that stratum with its 95% confidence interval, as also reported in Table 12. Note that the axis ranges differ between panels, since each panel is scaled to the range of the data in its own stratum.

**Figure 6:**
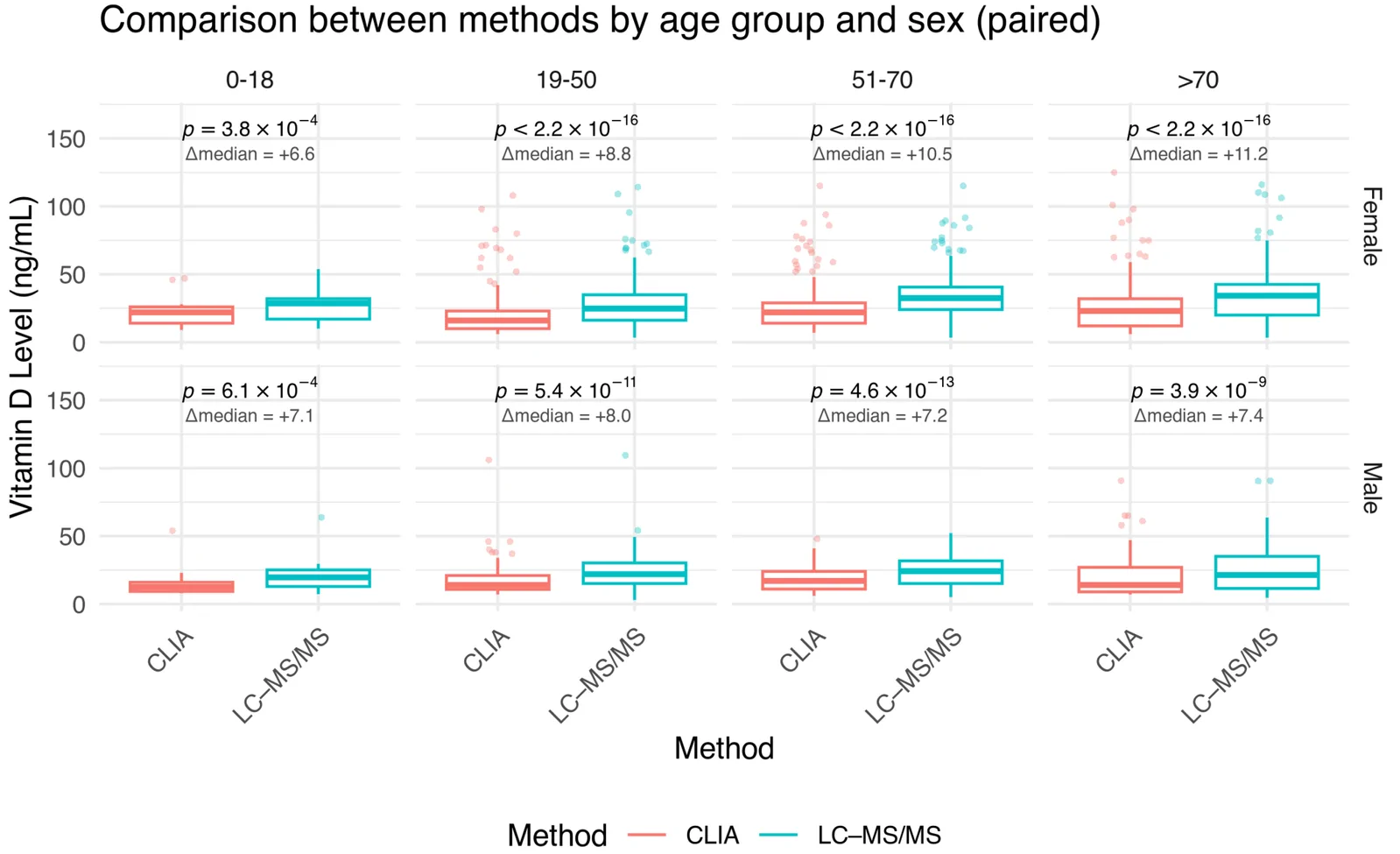
Comparison of the two methods by age group and sex. Each panel corresponds to one sex and age stratum; within each panel, each box shows the median and interquartile range of the values returned by one method, with whiskers extending to 1.5 times the interquartile range, and the dots above the whiskers are the individual values falling outside that range, spread horizontally so that overlapping samples remain distinguishable. The paired Wilcoxon *p*-value and the difference between the two medians are reported at the top of each panel.

Beyond the overall analysis, sex- and age-stratified comparisons are illustrated in Figure 5 and Figure 6, while the corresponding intraclass correlation coefficients and Passing–Bablok slopes are reported in Table 11 and Table 12. Stratified analyses confirmed statistically significant differences between the two measurement methods across all sex and age categories.

Breusch–Pagan tests performed within each stratum revealed significant heteroscedasticity in most adult groups (p<0.01), indicating that the variance of the differences increased with measurement magnitude. No significant heteroscedasticity was observed in paediatric strata; however, the limited sample size in these groups reduces statistical power (Table 13). Accordingly, the paediatric strata (n=17 and n=14) are retained for exploratory purposes only and do not support firm conclusions on assay performance within these subgroups.

Consistency-based ICC values remained uniformly high across strata (0.90–0.96), confirming strong rank-order preservation between methods regardless of demographic subgroup. In contrast, absolute agreement CCC Lin’s concordance correlation coefficient ($\rho_{c}$) were systematically lower (0.79–0.90), reflecting the proportional bias identified by Passing–Bablok regression rather than true measurement instability. Concordance metrics were generally stable across sex and age groups, suggesting that the systematic relationship between methods is demographically consistent. Confidence intervals were wider in paediatric groups owing to the limited number of observations.

### 3.3. Clinical Classification

**Figure 7:**
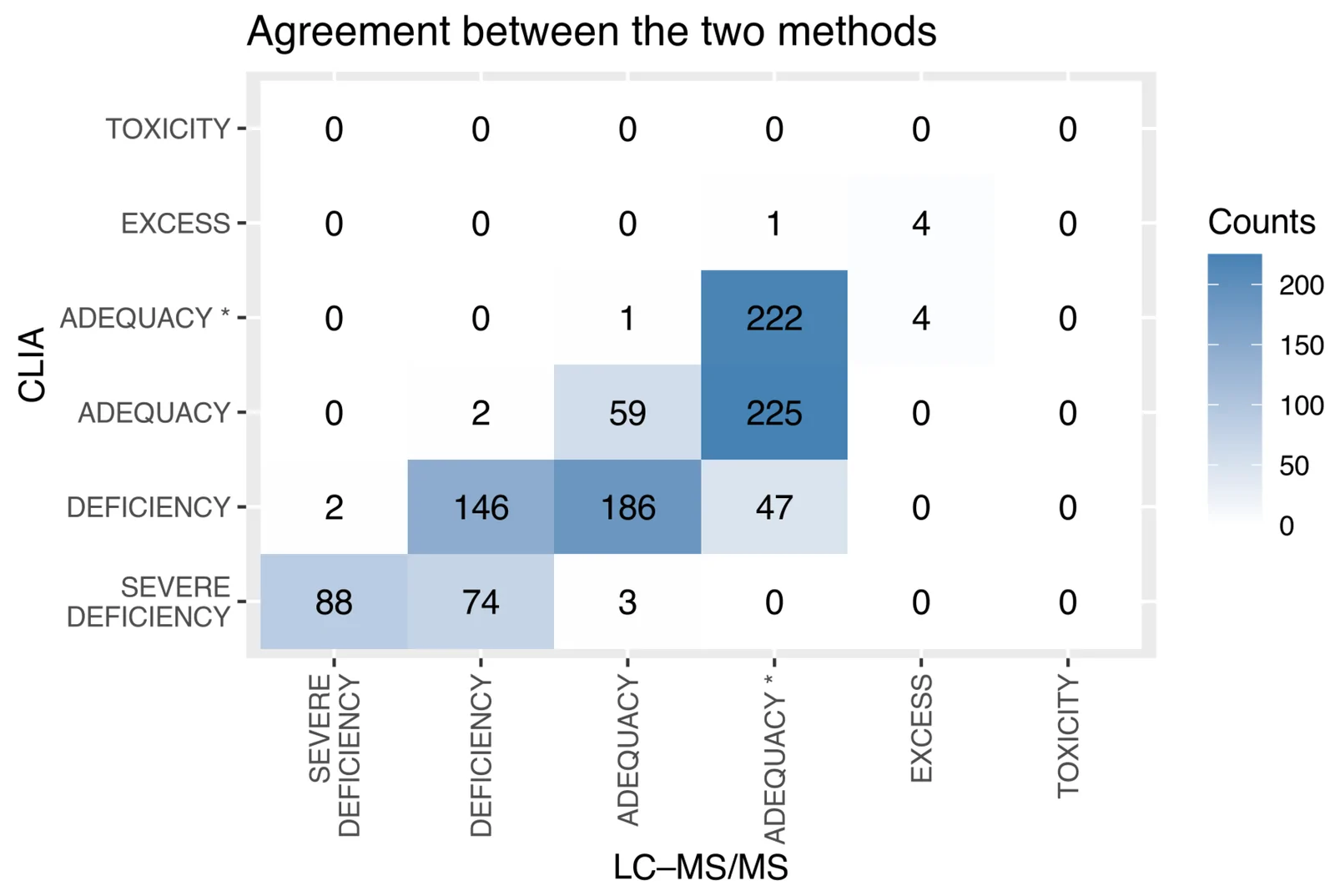
Six-class confusion matrix comparing the LC–MS/MS and CLIA classifications. The asterisk (*) marks ADEQUACY at optimal levels (30–100 ng/mL).

The studied population has been classified into six clinical risk categories (see Table 14 and Figure 7), agreeing with principal scientific and institutional sources [4,23].

The Cohen’s kappa test yielded an unweighted kappa of 0.32 with confidence interval (0.28, 0.36) and a weighted kappa of 0.72 with confidence interval (0.70, 0.75). The confidence intervals are narrow due to the study’s statistical power.

The unweighted kappa value (0.32) indicates weak-to-moderate agreement according to conventional scales (0.21–0.40 = “fair”) [38], meaning that the two methods classify samples in the same category with only a modest probability.

The weighted kappa (0.72), on the other hand, indicates good-to-substantial agreement (0.61–0.80 = “substantial”) [38], meaning that although the two methods do not always classify samples in exactly the same category, the discrepancies between their classifications tend to be limited; that is, differences often occur between adjacent categories. This has a clinical impact. Specifically, an adjacent-category shift across a decision threshold reclassifies a patient’s vitamin D status between the two platforms and can change the supplementation decision, reducing the number of individuals classified as requiring treatment; the two platforms therefore yield a different, not an erroneous, classification, and the high weighted agreement confirms that the clinically more consequential non-adjacent misclassifications are rare.

**Figure 8:**
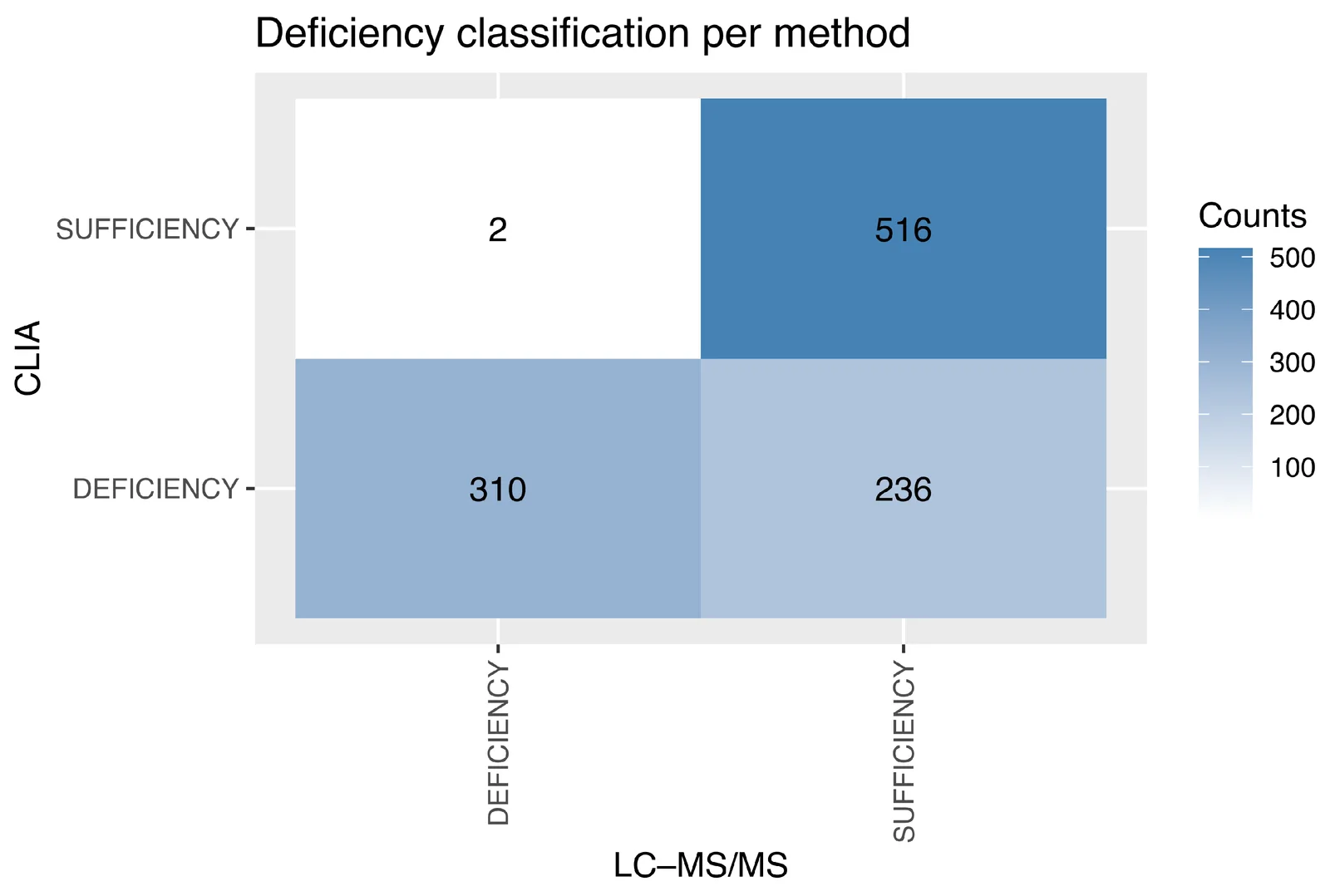
Two-class confusion matrix comparing the LC–MS/MS and CLIA classifications (deficiency vs. sufficiency).

By reducing the classification to two classes as in Figure 8, Cohen’s kappa changes, indicating moderate agreement, with an unweighted kappa of 0.56 (CI 0.51–0.60); with only two categories, differences can occur only between adjacent categories.

LC–MS/MS vs CLIA identify DEFICIENCY (29.32% vs. 51.32%) and SUFFICIENCY (69.62% vs. 48.21%) differently.

Moreover, McNemar’s test conducted on the deficiency classification between the two methods shows statistical significance ($p<2.2\times 10^{-16}$), indicating a significant difference in the clinical classification of the two methods.

The discordance ratio quantifies the asymmetry between the two methods’ classifications: DR=b/c=236/2=118. This indicates a strong directional imbalance in discordant classifications: among discordant samples, the CLIA method classifies a sample as deficient rather than sufficient 118-fold more often when the LC–MS/MS method classifies it as sufficient rather than deficient.

## 4. Discussion

Beyond its classical role in calcium–phosphate homeostasis and bone metabolism, vitamin D has been proposed to exert pleiotropic effects through modulation of gene transcription in multiple tissues, including cardiovascular, immune, and endocrine systems. These hypotheses have been supported by in vitro and observational data suggesting potential protective effects against cancer, cardiovascular disease, immune and autoimmune disorders, infections, and diabetes [39].

However, the results of several large randomised trials (RCTs) have not fully confirmed a causal relationship between vitamin D supplementation and the reduction of disease risk for the aforementioned conditions, especially in vitamin D-replete patients. The interpretation of these RCTs’ results warrants caution: most RCTs enrolled participants with adequate baseline 25-hydroxyvitamin D [25(OH)D] levels, thereby limiting the ability to detect benefits in deficient individuals. Observational data consistently indicate a threshold effect, with benefits potentially confined to concentrations below approximately 20 ng/mL. Heterogeneity in dosing regimens—ranging from daily physiological doses to intermittent high boluses—introduces further variability in outcomes, as bolus administration may induce transient non-physiological peaks with uncertain immunologic effects. Furthermore, differences in assay standardisation for serum 25(OH)D measurement across trials may have contributed to misclassification of vitamin D status, influencing interpretation of subgroup analyses [40].

From a clinical laboratory perspective, accurate measurement of 25(OH)D remains critical for identifying true deficiency, guiding supplementation, and interpreting research outcomes. Despite the lack of strong evidence supporting extraskeletal benefits in the general population, maintaining adequate vitamin D status remains essential for skeletal health and may have selective benefits in deficient or high-risk populations. Consensus statements and clinical guidelines from major endocrinology and nutrition societies emphasise that vitamin D testing and supplementation should focus on individuals at very high risk of severe deficiency (older and institutionalised adults, individuals with limited sun exposure due to geographic location, patients with malabsorption syndromes, obesity, osteoporosis, liver diseases, women during pregnancy or lactation, infants and children) rather than on the general population [41,42].

Inter-assay variability, lack of harmonisation and differences in reference materials have led to systematic bias and misclassification of vitamin D status across studies, potentially obscuring true associations and subgroup effects. The Vitamin D Standardisation Program (VDSP) and the development of certified reference materials (SRMs) by NIST provide tools for retrospective standardisation and prospective assay certification, improving comparability of 25(OH)D results across laboratories and time [30,32].

A recent consensus statement published in Endocrine Reviews [41] provides a comprehensive examination of the principal controversies inherent to the complex pathophysiological and clinical framework of the “Vitamin D system”, encompassing its metabolism, analytical assessment, biological actions, and therapeutic supplementation. The document aims to delineate the potential relevance of vitamin D across diverse clinical scenarios and to furnish methodologically robust evidence to address three fundamental questions (‘why, when, and how’) about the evaluation of vitamin D status and the rational use of supplementation, thereby supporting forthcoming revisions of international recommendations.

In the comparison between immunoassay-based methods and mass spectrometry techniques—an issue extensively debated in the last years in the international literature [43,44]—the measurement of vitamin D by mass spectrometry, despite some persisting limitations, currently represents the methodological gold standard [24,26,45,46].

This approach provides the clinician with analytically robust and clinically essential information for safeguarding individual health and for supporting well-considered decisions regarding preventive strategies, pharmacological treatment, and effective therapeutic monitoring.

In general, data from the international VDSP indicate that LC–MS/MS methods—whether developed as laboratory-developed tests (LDTs) or manufactured as commercial assays—demonstrate superior analytical performance compared with immunometric techniques. In particular, in terms of the ‘Individual Pass Rate’ indicator, the comparative evaluation (summarised in Table 15) of the vitamin D assays obtained using the two analytical platforms assessed in the present study shows that the Thermo Scientific Cascadion SM Clinical Analyser exhibits different performance than the Siemens Atellica IM 1600 Analyser. The same source further underscores that this issue is not solely attributable to the specific methodology or analytical platform used in our study but is a general characteristic of immunometric technologies.

Three independent studies [47,48,49] previously evaluated the analytical performance of the Cascadion SM Clinical Analyser against commonly used immunometric methods and in-house LC–MS/MS assays. Benton et al. compared Cascadion with three methods in 146 patient samples, showing a marked overestimation by Cascadion versus the Siemens ADVIA Centaur (+34%) and substantial agreement versus the Roche Cobas (approximately +2%). Junger et al. reported a slight overestimation of Cascadion relative to the Abbott Alinity immunoassay (+6.6%). Gqamana et al. compared Cascadion with the Roche Cobas e602 using 120 CDC-certified samples together with routine samples, and observed a mean overestimation by Roche relative to Cascadion in samples with normal triglyceride levels. These findings confirm that the discordances between LC–MS/MS and immunometric platforms depend on features of the technologies rather than being specific to the platform assessed here. Concordantly, a recent comparison on different platforms (Roche electrochemiluminescence immunoassay versus an independent LC–MS/MS assay) likewise found LC–MS/MS to yield systematically higher 25(OH)D concentrations than the immunoassay, with method-dependent reclassification of a subset of subjects around the clinical decision thresholds [37].

We included a large study cohort, which ensured substantial statistical power and enabled multiple comparisons while maintaining adequate analytical robustness. With 1064 paired samples distributed across eight pre-specified sex- and age-based strata, the present cohort is substantially larger than previous 25(OH)D method-comparison studies, which have generally been limited to a few hundred paired measurements or fewer—for instance, the recent CLIA versus LC–MS/MS comparison of [37] analysed 138 samples with a 59-sample validation set. This numerosity affords the statistical power required to resolve the concentration-dependent bias and the demographic modulation of inter-method agreement that smaller, hypothesis-generating studies are underpowered to establish. We designed the study to entail a comparative assessment of the two methodologies across several age categories, further stratified by sex; such stratification not only mitigates the potential confounding effects attributable to age and sex but also demonstrates that the inter-method bias manifests with noteworthy consistency and coherence across all strata examined. Although the primary analysis employed the Wilcoxon paired-sample test—already controlling for intra-individual variability—the additional stratified analyses provide a critical incremental value, demonstrating that the observed effect is not restricted to a specific subgroup but is uniformly evident across the entirety of the demographic categories considered. This methodological rigour not only strengthens the internal validity of the findings but also enhances their external applicability, highlighting the pervasive and systematic nature of the phenomenon under investigation.

These considerations must furthermore be contextualised within the broader epidemiological landscape, in which vitamin D deficiency represents a globally prevalent condition affecting large segments of the population. Factors such as age, sex, skin pigmentation, dietary and nutritional habits, levels of physical activity, patterns of sun exposure, and associated comorbidities collectively contribute to an elevated risk of moderate to severe hypovitaminosis in many individuals [50]. Method-specific interferences also distribute along the age and sex axes: the C3-epimer of 25(OH)D_3_, handled differently by immunoassays and LC–MS/MS, is present at higher fractions in infants and declines with age [51], while standardisation of immunoassay values against LC–MS/MS has been shown to narrow an apparent sex gap in deficiency rates, indicating that part of that gap is an assay artefact [52]. These observations, together with the reported variation of 25(OH)D levels by age, sex, lifestyle and supplement use [53], motivate our pre-specified stratification of the method comparison by sex and age, the only demographic variables available to us, as a sensitivity analysis confirming that the systematic bias is homogeneous across clinically relevant subpopulations. Consistent with this rationale, a recent CLIA versus LC–MS/MS comparison incorporating age and sex into a fuzzy-inference model reported that the inter-method discordance was concentrated in a specific demographic subgroup (women aged 30–40 years)—a signal the authors qualified as hypothesis-generating and requiring confirmation in larger cohorts with multivariable adjustment [37]; our adequately powered, pre-specified stratification directly addresses that call.

In our study, the analytical limitations characterising immunometric platforms, as evidenced by the heteroscedastic and non-linear bias structure described above, preclude the derivation of a reliable calibration formula from Passing–Bablok regression (y=1.33x+1.01): the concentration-dependent, non-linear heteroscedasticity indicates that the relationship between methods is not stable across the measurement range, and the residual limits of agreement would remain critical even after bias correction, given that therapeutic decision thresholds for 25-OH vitamin D are separated by as little as 10–20 ng/L. For laboratories unable to adopt LC–MS/MS immediately, harmonisation through external quality assessment schemes (e.g., DEQAS, NIST SRM 972a) represents a more appropriate interim strategy.

Within this framework, our analysis—conducted both on the overall cohort and through age- and sex-stratified sub-analyses—suggests that the use of LC–MS/MS technology may permit a clinically significant reclassification of vitamin D status, with a shift from insufficiency or severe insufficiency toward concentrations deemed sufficient. In routine clinical laboratory practice, such a reclassification of the general population, particularly in the context of assessing the need for therapeutic supplementation, may also entail meaningful economic implications by potentially reducing the number of individuals requiring vitamin D treatment.

## Figures and Tables

**Table 1:** Cascadion calibration (lot 73726350).

	Vitamin D_2_ (ng/mL)	Vitamin D_3_ (ng/mL)
Cal 1	3.6	4.2
Cal 2	7.5	8.3
Cal 3	15.3	16.3
Cal 4	31.2	32.3
Cal 5	62.5	64.5
Cal 6	115.9	121.4

**Table 2:** Atellica calibration (lots C3432, C3440).

Lot	C3432 (ng/mL)	C3440 (ng/mL)
Low	19.8	23
High	110	119

**Table 3:** Three lots of quality control materials used on the Cascadion platform for vitamin D_2_.

	Target Value (ng/mL)	No. Samples	Mean (ng/mL)	SD (ng/mL)	CV%
CONTROL Level 1 Lot 73726351	9.4	68	9.87	0.55	5.6
CONTROL Level 2 Lot 73726354	28.5	68	29.53	1.26	4.1
CONTROL Level 3 Lot 73726355	84.8	68	87.72	2.90	3.3

**Table 4:** Three lots of quality control materials used on the Cascadion platform for vitamin D_3_.

	Target Value (ng/mL)	No. Samples	Mean (ng/mL)	SD (ng/mL)	CV%
CONTROL Level 1 Lot 73726351	10.4	68	10.6	0.5	4.2
CONTROL Level 2 Lot 73726354	29.8	68	30.9	1.3	4.1
CONTROL Level 3 Lot 73726355	88.7	68	91.2	3.0	3.3

**Table 5:** Two lots of quality control materials used on the Atellica platform for total 25(OH)D.

	Target Value (ng/mL)	No. Samples	Mean (ng/mL)	SD (ng/mL)	CV%
CONTROL Level 1 Lot 60270T	33.00	11	35.55	1.40	3.9
CONTROL Level 1 Lot 64920T	34.00	60	31.30	2.78	8.9
CONTROL Level 2 Lot 60270T	85.90	11	87.75	3.00	3.4
CONTROL Level 2 Lot 64920T	84.50	60	81.38	3.64	4.5

**Table 6:** Statistical distribution of patient age in the total laboratory population (*n* = 12,404) requiring vitamin D essay.

Patients	F	M	Total
Number	9704	2700	12,404
%	78.23	21.77	100.00
**Age (years)**	**F**	**M**	**Total**
IQR (Q3−Q1)	21	28	26
Mean	62	62	62
SD	18	20	18
Min	4	4	4
Max	111	101	111
2.5th percentile	24	16	21
50th percentile	61	65	62
97.5th percentile	92	92	92

**Table 7:** Sample distribution by age group, total laboratory population requiring vitamin D essay.

Age (Years)	F	M	Total
0–18	107	102	209
19–50	2358	583	2941
51–70	3950	948	4898
>70	3289	1067	4356
**Total**	9704	2700	12,404

**Table 8:** Study population characteristics (selected sub-cohort, *n* = 1064).

	Total	Female	Male
N (%)	1064 (100)	828 (77.8)	236 (22.2)
**Age (years)**			
Mean (SD)	58 (19)	58 (18)	57 (21)
Median (IQR)	59 (28)	58 (33)	61 (47)
Range (min–max)	4–97	7–96	4–97
2.5th–97.5th percentile	17–87	21–87	11–86
**Age group, N (%)**			
0–18	33 (3.1)	18 (2.2)	15 (6.4)
19–50	330 (31.0)	261 (31.5)	69 (29.2)
51–70	369 (34.7)	297 (35.9)	72 (30.5)
>70	332 (31.2)	252 (30.4)	80 (33.9)

**Table 9:** Total 25(OH)D in females. Concentrations are reported in ng/mL and nmol/L.

Statistic	LC–MS/MS (ng/mL)	LC–MS/MS (nmol/L)	CLIA (ng/mL)	CLIA (nmol/L)
2.5th percentile	6.21	15.53	7.00	17.50
50th percentile	30.93	77.33	20.00	50.00
97.5th percentile	74.85	187.13	71.00	177.50
IQR	20.16	50.40	17.00	42.50
Min	3.40	8.50	6.00	15.00
Max	116.00	290.00	125.00	312.50

**Table 10:** Total 25(OH)D in males. Concentrations are reported in ng/mL and nmol/L.

Statistic	LC–MS/MS (ng/mL)	LC–MS/MS (nmol/L)	CLIA (ng/mL)	CLIA (nmol/L)
2.5th percentile	5.19	12.98	7.00	17.50
50th percentile	21.92	54.80	15.00	37.50
97.5th percentile	55.73	139.33	54.50	136.25
IQR	17.85	44.63	14.00	35.00
Min	3.00	7.50	6.00	15.00
Max	109.40	273.50	106.00	265.00

**Table 11:** Intraclass correlation coefficients for consistency (ICC(C,1)), and Lin’s concordance correlation coefficient (CCC) stratified by sex and age group. Values are reported with 95% confidence intervals.

Sex	Age Group	*n*	ICC(C,1)	CCC
			(95% CI)	(95% CI)
F	0–18	17	0.94 (0.83–0.98)	0.85 (0.68–0.93)
F	19–50	244	0.92 (0.90–0.94)	0.83 (0.80–0.86)
F	51–70	300	0.94 (0.92–0.95)	0.80 (0.77–0.83)
F	>70	267	0.92 (0.91–0.94)	0.83 (0.80–0.86)
F	Overall	828	0.93 (0.92–0.94)	0.83 (0.81–0.84)
M	0–18	14	0.95 (0.86–0.99)	0.87 (0.71–0.94)
M	19–50	64	0.96 (0.94–0.98)	0.90 (0.85–0.93)
M	51–70	77	0.90 (0.85–0.93)	0.79 (0.71–0.85)
M	>70	81	0.92 (0.88–0.95)	0.88 (0.83–0.92)
M	Overall	236	0.93 (0.91–0.94)	0.87 (0.84–0.89)
Overall	Overall	1064	0.93 (0.92–0.94)	0.84 (0.82–0.85)

**Table 12:** Passing–Bablok regression intercept and slope with 95% confidence intervals, stratified by sex and age group. * Confidence interval excludes 0 for intercept and 1 for slope.

Sex	Age Range	Sample Count	Passing–Bablok Intercept (95% CI)	Passing–Bablok Slope (95% CI)
F	0–18	17	2.07 (−4.81–6.02)	1.16 (0.92–1.49) *
F	19–50	244	0.39 (−1.44–2.04)	1.40 (1.29–1.52) *
F	51–70	300	4.94 (3.02–6.51) *	1.19 (1.12–1.28) *
F	>70	267	1.53 (−0.34–3.76)	1.31 (1.21–1.41) *
F	Overall	828	2.12 (1.02–3.06) *	1.30 (1.24–1.36) *
M	0–18	14	−1.16 (−11.96–4.37)	1.53 (1.11–2.42) *
M	19–50	64	−1.02 (−4.13–2.96)	1.38 (1.17–1.64) *
M	51–70	77	−0.67 (−3.22–2.16)	1.31 (1.14–1.53) *
M	>70	81	−2.91 (−5.61–0.35)	1.44 (1.24–1.67) *
M	Overall	236	−1.36 (−3.16–0.37)	1.38 (1.26–1.51) *
Overall	Overall	1064	1.01 (0.01–1.86) *	1.33 (1.28–1.38) *

**Table 13:** Breusch–Pagan tests for heteroscedasticity of method differences, stratified by sex and age group.

Sex	Age Group	*n*	BP Statistic	*p*-Value
F	0–18	17	1.63	$2.02\times 10^{-1}$
F	19–50	244	55.4	$9.94\times 10^{-14}$
F	51–70	300	25.7	$3.92\times 10^{-7}$
F	>70	267	32.4	$1.28\times 10^{-8}$
M	0–18	14	0.191	$6.62\times 10^{-1}$
M	19–50	64	6.51	$1.07\times 10^{-2}$
M	51–70	77	13.5	$2.34\times 10^{-4}$
M	>70	81	25.7	$3.95\times 10^{-7}$

**Table 14:** Comparison of classifications between the LC–MS/MS and CLIA methods. Concentration limits are reported in ng/mL and nmol/L (conversion factor: 1 ng/mL = 2.5 nmol/L).

Classification	Limits	Limits	LC–MS/MS		CLIA	
	(ng/mL)	(nmol/L)	N	%	N	%
SEVERE DEFICIENCY	<10	<25	90	8.46	165	15.51
DEFICIENCY	10–19	25–47.5	222	20.86	381	35.81
SUFFICIENCY (non-optimal)	20–30	50–75	249	23.40	286	26.88
SUFFICIENCY (optimal)	31–100	77.5–250	495	46.52	227	21.33
EXCESS	101–150	252.5–375	8	0.75	5	0.47
TOXICITY	>150	>375	0	0.00	0	0.00
**Total**	–	–	1064	100	1064	100

**Table 15:** Vertical comparison of platform performance in the Vitamin D Standardisation Program.

Attribute	Siemens Healthineers	Thermo Fisher Scientific
**Principle**	Chemiluminescence Immunoassay	LC–MS/MS †
**Assay Identifier**	Atellica^®^ IM Vitamin D Total assay	Cascadion^®^ SM 25-Hydroxy Vitamin D
**Individual Samples Pass Rate (%)**		
Q1 2021	28	80
Q2 2021	18	80
Q3 2021	36	82

† Considered the reference method.

## Data Availability

The data and the analysis code (R version 4.6.0 and Python version 3.13.7 scripts) generated during this study are available from the corresponding author on reasonable request. They are not publicly available owing to patient privacy restrictions.
